# Defining the HLA class I‐associated viral antigen repertoire from HIV‐1‐infected human cells

**DOI:** 10.1002/eji.201545890

**Published:** 2015-11-04

**Authors:** Nicola Ternette, Hongbing Yang, Thomas Partridge, Anuska Llano, Samandhy Cedeño, Roman Fischer, Philip D. Charles, Nadine L. Dudek, Beatriz Mothe, Manuel Crespo, William M. Fischer, Bette T. M. Korber, Morten Nielsen, Persephone Borrow, Anthony W. Purcell, Christian Brander, Lucy Dorrell, Benedikt M. Kessler, Tomáš Hanke

**Affiliations:** ^1^ The Jenner InstituteNuffield Department of MedicineUniversity of OxfordOxfordUK; ^2^ Target Discovery InstituteNuffield Department of MedicineUniversity of OxfordOxfordUK; ^3^ NIHR Oxford Biomedical Research CentreOxfordUK; ^4^ Nuffield Department of MedicineUniversity of OxfordOxfordUK; ^5^ HIVACAT, Irsicaixa AIDS Research InstituteAutonomous University of BarcelonaBadalonaSpain; ^6^ Department of Biochemistry and Molecular BiologyMonash UniversityClaytonVictoriaAustralia; ^7^ Lluita contra la Sida’ FoundationHospital Germans Trias i PujolBadalonaSpain; ^8^ Universitat de Vic – Universitat Central de CatalunyaVicSpain; ^9^ HIV Unit, Hospital de la Vall d'HebrónBarcelonaSpain; ^10^ Group T‐6, Theoretical BiologyLos Alamos National LaboratoryLos AlamosNMUSA; ^11^ Department of Systems BiologyCenter for Biological Sequence AnalysisTechnical University of DenmarkKongens LyngbyDenmark; ^12^ Institució Catalana de Recerca i Estudis Avançats (ICREA)BarcelonaSpain

**Keywords:** Cytotoxic T cells, Human immunodeficiency virus type I, Human leukocyte antigen, Immunopeptidome, Mass spectrometry

## Abstract

Recognition and eradication of infected cells by cytotoxic T lymphocytes is a key defense mechanism against intracellular pathogens. High‐throughput definition of HLA class I‐associated immunopeptidomes by mass spectrometry is an increasingly important analytical tool to advance our understanding of the induction of T‐cell responses against pathogens such as HIV‐1. We utilized a liquid chromatography tandem mass spectrometry workflow including de novo‐assisted database searching to define the HLA class I‐associated immunopeptidome of HIV‐1‐infected human cells. We here report for the first time the identification of 75 HIV‐1‐derived peptides bound to HLA class I complexes that were purified directly from HIV‐1‐infected human primary CD4^+^ T cells and the C8166 human T‐cell line. Importantly, one‐third of eluted HIV‐1 peptides had not been previously known to be presented by HLA class I. Over 82% of the identified sequences originated from viral protein regions for which T‐cell responses have previously been reported but for which the precise HLA class I‐binding sequences have not yet been defined. These results validate and expand the current knowledge of virus‐specific antigenic peptide presentation during HIV‐1 infection and provide novel targets for T‐cell vaccine development.

## Introduction

Cytotoxic T lymphocyte (CTL) mediated recognition and elimination of infected cells is a major arm of the immune response against intracellular pathogens 1. Typically, CTLs are CD8+ T lymphocytes, which recognize virus‐derived peptides presented on the surface of infected cells in complex with HLA class I molecules 2, 3. Aside from innate and humoral responses, induction of effective CTL responses by vaccination is likely required for protection particularly against pathogens that replicate intracellularly and for which induction of sterilizing immunity is difficult. Examples include the causative agents of malaria, tuberculosis, and acquired immunodeficiency syndrome (HIV/AIDS).

HIV/AIDS continues to be a major global health problem 4. There is strong evidence that CD8^+^ T cells contribute to the control of acute and chronic HIV‐1 infection in a major way 5. Understanding the characteristics of the HLA class I‐associated peptidomes on the surface of HIV‐1‐infected cells has the potential to crucially inform the development of effective preventive and therapeutic T‐cell vaccines. Such improved understanding may also provide further insights into allele‐specific binding motifs and more general phenomena such as the protective role of certain HLA alleles 5 and factors that define T‐cell immunodominance 6.

Recent advances in the technology of nanoflow liquid chromatography tandem mass spectrometry (LC‐MS/MS) allow the direct qualitative evaluation of HLA‐associated peptidomes 7, 8. In the context of HIV‐1 infection, cells overexpressing individual viral proteins were analyzed for presentation of viral HLA‐associated peptides 9, 10 and recently, peptides were purified from soluble HLA‐A molecules secreted from a HIV‐1‐infected T‐cell line 11. In each case, viral antigen was either delivered by transfection of plasmids encoding selected HIV‐1 proteins or by continuous infection cycles of immortalized cells lines secreting soluble HLA molecules in bioreactors. Despite the limitations of these approaches, they yielded a number of important observations, including identification of a considerable number of previously unmapped, putative T‐cell epitopes, and highlighting the paucity of HIV‐1 peptides within the complex immunopeptidome of the HIV‐1‐infected cell.

Here, we used an immunopurification protocol to specifically isolate and identify a large number of peptides bound to HLA class I complexes from HIV‐1‐infected primary CD4^+^ T cells and C8166 cells (a cell line efficiently infected by HIV‐1) utilizing an LC‐MS/MS analysis workflow.

## Results

### Characterization of the HLA class I‐associated immunopeptidome of HIV‐1‐infected cells

To prepare infected cells, the human immortalized cell line C8166 (A*01:01/01:01, B*08:01/44:02; C*05:01/07:01) or primary CD8^+^ cell‐depleted PBMC from three HIV‐1‐uninfected individuals (C6 of A*24:02/29:02, B*35:03/45:01, C*04:01/06:02; C7 of A*11:01/68:01, B*07:02/27:05, C*01:02/07:02; and C8 of A*29:02/30:04, B*41:01/44:03, C*16:01/17:01 genotypes) was optimally infected with HIV‐1 IIIB at a multiplicity of infection (MOI) that yielded maximal infection rates of 68.2% for the cell line and 19.8, 21.3, and 22.3% for the primary cell samples C6, C7, and C8, respectively. Infection was estimated by an intracellular anti‐p24 antigen staining 12, and approximately 10^8^ infected cells were used per analysis. As negative control, uninfected C8166 cells and CD8^+^ cell‐depleted PBMC from C8 were analyzed in parallel. Cells were lysed and a resin‐linked, HLA class I‐specific, conformation‐dependent monoclonal antibody W6/32 was used to capture peptide‐loaded HLA complexes. Noncovalent interactions among the complex components were abolished by acid treatment, eluted peptides were separated by reverse‐phase HPLC from the α‐chain and β_2_‐microglobulin of the HLA complexes and the eluted peptide fractions were analyzed by LC‐MS/MS. Collected spectra were interpreted using PEAKS and MASCOT utilizing a protein database that included translations of all the six open reading frames of the complete genomic sequence and annotated protein sequences of the HIV‐1 IIIB stock used for infections.

**Figure 1:**
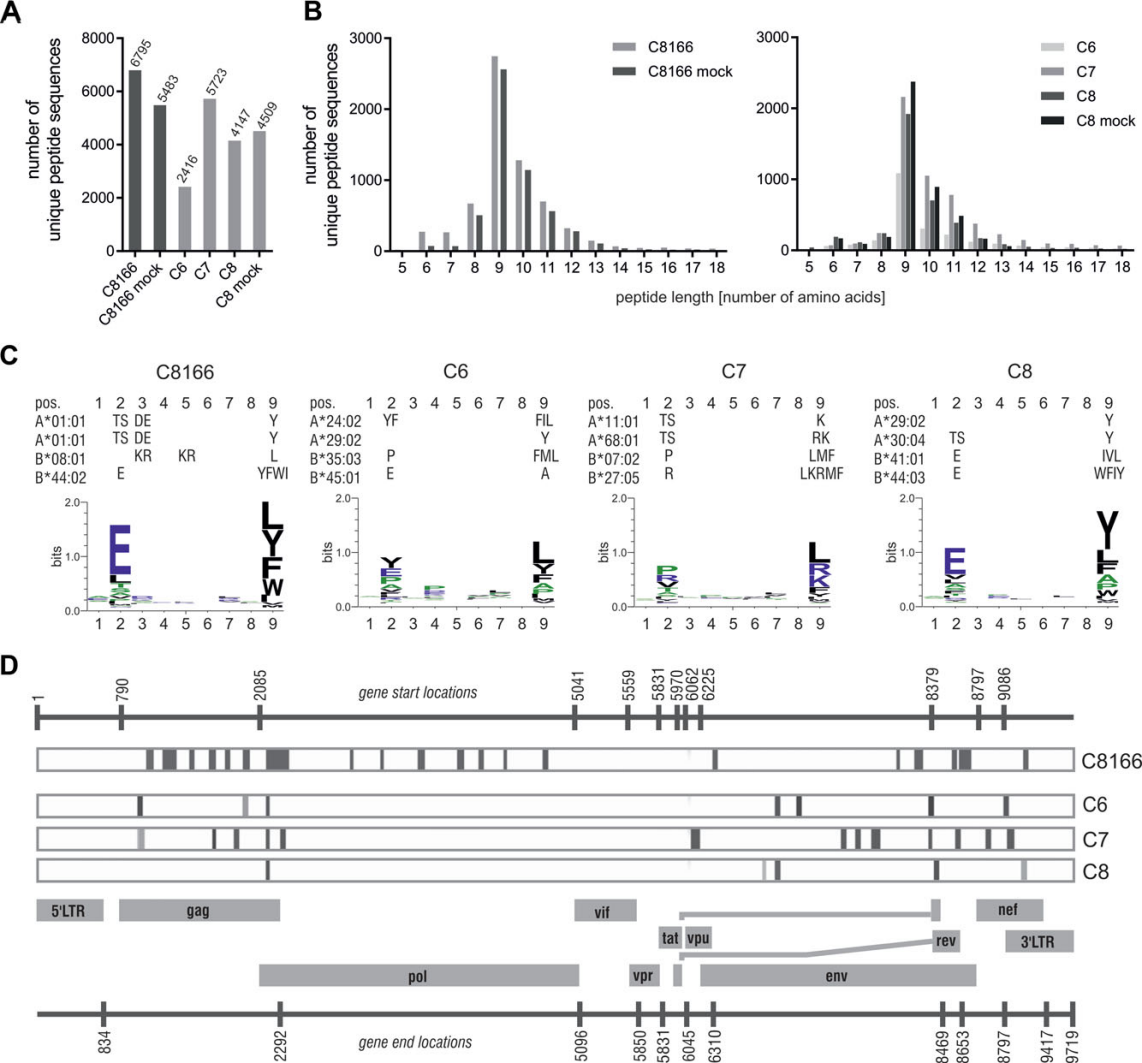
HLA class I‐associated peptides from HIV‐1‐infected cells. The human T‐cell line C8166 and primary CD4^+^ cells from three individuals (samples C6, C7, C8) were infected with HIV‐1 for 5–7 days. HLA class I‐associated peptides were purified and analyzed using LC‐MS/MS. (A) The total numbers of unique peptide sequences identified by LC‐MS/MS in each sample from a single immunopecipitation experiment with W6/32 antibody and (B) the length distributions of identified peptides in the C8166 cell line (left) and primary CD4^+^ cells (right) are shown. (C) Motif analysis of all eluted 9‐mer peptides for each of the HIV‐1‐infected samples (Weblogo 3.4 17). Known anchor residues for the relevant HLA‐A and HLA‐B subtypes are listed above the graphs for each sample (information from MHC Motif Viewer 37, 38). The size of the letter representing the amino acid in the indicated position is scaled according to the frequency of occurrence in the peptide. (D) Schematic overview of all HIV‐1‐derived immunopeptides identified in the samples relative to the position of the HIV‐1 proteins assigned within the viral genome, which are depicted as gray boxes. Numbers above and below the gray bars indicate the nucleotide position of the starts and ends of the regarding HIV‐1 genes using the strain HXB2 annotation (generated using the Los Alamos National Laboratory HIV Sequence Locator Tool). The position of each identified peptide sequence relative to the position in the HXB2 annotation is indicated as vertical gray line in the rectangular panel depicted for each sample.

A range between 2416 and 6795 unique peptides was identified in the C8166 and C6, C7, and C8 cell samples (Fig. 1A), of which 75 unique peptides (1.1%) in total were derived from HIV‐1 (Table 1); no HIV‐1‐derived peptides were identified in mock‐infected cells. Although the peptides ranged from 5 to 52 amino acids in length, 78–92% of peptides were 8–12 amino acids long and 9‐mers were overall the most abundant species (Fig. 1B). Long peptides could be originated from HLA complexes in the ER, which are estimated to be 5–10% of the total HLA complex population of the cell 13. In addition, HLA‐bound peptides longer than 12 and up to 25 amino acids have been characterized in the context of several HLA class I alleles 14, 15, 16.

**Table 1:** HLA class I‐associated peptides eluted from HIV‐1 IIIB‐infected cells

Name	Peptide	Sample	HXB2	Predicted binding	IC50	Rank	Predicted	Reported epitope	Reported HLA allele	PEAKS^f^	MASCOT^f^	SM^g^	ELISPOT^h^
			[aa]^a^	segment^b^	[nM]	[%]^c^	HLA allele	(LANL‐HIVDB)^d^	(LANL‐HIVDB)^e^				
AF8	ASRELERF	6	Gag (37‐44)	ASRELERF	17 110	10.00	C*04:01	ASRELERF	B*35;01	23		+	5000
AA9	ASRELERFA	6	Gag (37‐45)	ASRELERF	17 110	10.00	C*04:01	HIVWASRELERFAVNPSL	C*04;01	19		+	None
AA11	AEAMSQVTNSA	6	Gag (364‐374)	AEAMSQVTNSA	36	0.05	B*45:01	AEAMSQVTNS	B*45;01	39		+	120
FY9	FLGKIWPSY	6; 7; 8	Gag (433‐441)	FLGKIWPSY	18	0.30	A*29:02	FLGKIWPSYK	A*02;01	54	31	+	230
FI9	FSNSAKSII	6	gp160 (277‐285)	FSNSAKSI	5607	4.00	C*06:02			27		+	60
SY9	SFEPIPIHY	6; 8	gp160 (209‐217)	SFEPIPIHY	48	0.80	A*29:02	SFEPIPIHY	A*29;02	22		+	120
AL10	AEGGIISLNL	6	gp160 (688‐697)	AEGGIISL	692	1.50	B*45:01			25		+	60
AP9	EEVGFPVTP	6	Nef (64‐72)	EEVGFPVT	569	1.00	B*45:01			27		+	None
AE16	ASRELERFAVNPGLLE	7	Gag (37‐52)	FAVNPGLL	1676	0.20	C*01:02	ERFAVNPGLL	B*27;01	30	37	+	680
SE15	SRELERFAVNPGLLE	7	Gag (38‐52)	FAVNPGLL	1676	0.20	C*01:02	ERFAVNPGLL	B*27;01	19		+	740
IK7	IILGLNK	7	Gag (266‐272)	na	na	0.00	na	KRWIILGLNK	B*27;01		38	+	550
LE11	LKALGAGATLE	7	Gag (334‐344)	KALGAGATL	2560	0.40	C*01:02			25		‐	70
ER9	ELYPLTSLR	7	Gag (482‐490)	ELYPLTSLR	8	0.15	A*68:01			33	30	+	90
QL10	QPIQIAIVAL	7	Vpu (2‐12)	QPIQIAIVAL	85	0.80	B*07:02	QPIQIAIAAL	B*07;02		37	+	None
VV12	VALVVAIIIAIV	7	Vpu (10‐21)	VALVVAIIIAI	13 373	7.00	C*01:02	VVAAIIAIV			29	‐	90
NQ10	NTRIPCRLKQ	7	gp160 (413‐422)	TRIPCRLK	289	0.80	B*27:05			24		na	60
NR10	NETNGTEIFR	7	gp160 (460‐469)	ETNGTEIFR	7	0.10	A*68:01			26		‐	60
RL8	RAAGITAL	7	gp160 (511‐518)	RAAGITAL	2011	0.25	C*01:02			23		+	None
LA10	LGAAGSAVGA	7	gp160 (523‐532)	LGAAGSAV	10 344	9.00	B*07:02			24		na	None
MV10	MLPLVIGAIV	7	gp160 (684‐693)	LPLVIGAI	125	0.80	B*07:02			22		na	1980
RR9	RDLVLIVTR	7	gp160 (772‐780)	DLVLIVTR	72	1.50	A*68:01			35		+	None
SR9	SVIGWPTVR	7	Nef (9‐17)	SVIGWPTVR	18	0.50	A*68:01	SVVGWPAVR	A03	25		+	60
QK10	QVPLRPMTYK	7	Nef (73‐82)	QVPLRPMTYK	89	1.00	A*11:01	QVPLRPMTYK	A*03;01; A11	19	25	+	2950
AK9	AVDLSHFLK	7	Nef (84‐92)	AVDLSHFLK	13	0.12	A*11:01	AVDLSHFLK	A*03;01; A11	35	38	+	1240
FS8	FLGKIWPS	8	Gag (433‐440)	FLGKIWPS	16 667	8.00	A*30:04	FLGKIWPS	A*02;01	27		na	na
VF8	VQKEYAFF	8	gp160 (169‐176)	VQKEYAFF	1236	0.50	A*30:04			19		na	na
VY9	VQKEYAFFY	8	gp160 (169‐177)	VQKEYAFFY	148	0.01	A*30:04			30		na	na
IY9	IVNRVRQGY	8	gp160 (704‐712)	IVNRVRQGY	238	0.01	A*30:04	IVNRVRQGY	A30	22		na	na
GY9	GYFPDWQNY	8	Nef (119‐127)	GYFPDWQNY	347	0.05	A*30:04	GYFPDWQNY	A24	44	47	na	na
QN18	QLQPSLQTGSEERRSLYN	C8166	Gag (63‐80)	GSEERRSLY	197	0.20	A*01:01	GSEELRSLY	A*01;01		25	na	None
SY22	SKKKAQQAAADTGHSSQVSQNY	C8166	Gag (111‐132)	AADTGHSSQV	99	0.25	C*05:01	KTQQAAADK; NSSKVSQNY	B57; B*35;01		40	na	None
TY11	TGHSSQVSQNY	C8166	Gag (122‐132)	HSSQVSQNY	278	0.25	A*01:01	DTGHSNQVSQNY	A33	16	40	+	5000
HY9	HSSQVSQNY	C8166	Gag (124‐132)	HSSQVSQNY	278	0.25	A*01:01	NSSKVSQNY	B*35;01	33	20	+	None
PA22	PIVQNIQGQMVHQAISPRTLNA	C8166	Gag (133‐154)	MVHQAISPRTL	1461	1.50	C*07:01	QAISPRTL	Cw*07	51		+	1145
MI8	MQMLKETI	C8166	Gag (198‐205)	MQMLKETI	923	2.00	B*08:01	AMQMLKETI	A2	29	32	+	4380
VK15	VGEIYKRWIILGLNK	C8166	Gag (258‐272)	EIYKRWIIL	149	0.50	B*08:01	EIYKRWII	B*08;01	22	27	na	5000
IK12	IYKRWIILGLNK	C8166	Gag (261‐272)	IYKRWIIL	3655	3.00	C*07:01	IYKRWIILGLNK	A24	28	23	na	1240
YK11	YKRWIILGLNK	C8166	Gag (262‐272)	YKRWIILGL	3984	3.00	C*07:01	IYKRWIILGLNK	A24	46	39	na	400
KK10	KRWIILGLNK	C8166	Gag (263‐272)	KRWIILGL	3478	3.00	C*07:01	KRWIILGLNK	B27	30	24	+	360
KI11	KRWIILGLNKI	C8166	Gag (263‐273)	KRWIILGL	3478	3.00	C*07:01	WIILGLNKI; IILGLNKI	na; A2, A3	20	33	+	670
WK8	WIILGLNK	C8166	Gag (265‐272)	WIILGLNK	27 259	32.00	A*01:01	KRWIILGLNK	B27	25	30	na	90
AW11	AEQASQEVKNW	C8166	Gag (306‐316)	AEQASQEVKNW	12	0.01	B*44:02	AEQASQEVKNW	B44, Cw5	75	68	+	4390
AW8	ASQEVKNW	C8166	Gag (309‐316)	ASQEVKNW	11 596	7.00	B*44:02	AEQASQEVKNW	B44, Cw5		64	+	1120
AM14	AEAMSQVTNSATIM	C8166	Gag (364‐377)	AEAMSQVT	1323	1.50	B*44:02	AEAMSQVTNS	B*45;01	34	31	+	60
SM10	SQVTNSATIM	C8166	Gag (368‐377)	VTNSATIM	1429	2.00	C*05:01	SQVTNSATI; QVTNSATIM	A2; na		34	na	80
FF16	FLGKIWPSYKGRPGNF	C8166	Gag (433‐448)	FLGKIWPSY	2677	1.50	A*01:01	FLGKIWPSYKGRPGN	A2	42	72	+	240
KF13	KIWPSYKGRPGNF	C8166	Gag (436‐448)	WPSYKGRPGNF	1249	3.00	B*08:01	KIWPSYKGR	A*3101	28	51	+	None
SQ12	SRPEPTAPPFLQ	C8166	Gag (451‐462)	SRPEPTAPPFL	199	0.20	C*07:01	EPTAPPEESF	B35, B58	21		+	None
SG16	SRPEPTAPPEESFRSG	C8166	Gag (451‐466)	PEESFRSG	17 531	10.00	B*44:02	EPTAPPEESF	B35, B58	57	48	+	410
EY17	ETTTPPQKQEPIDKELY	C8166	Gag (468‐484)	QEPIDKELY	4181	3.00	B*44:02	TPSQKQEPI	B35, B53	26	22	+	None
TY16	TTTPPQKQEPIDKELY	C8166	Gag (469‐484)	QEPIDKELY	4181	3.00	B*44:02	TPSQKQEPI	B35, B53	48	35	+	70
PP13	PLTSLRSLFGNDP	C8166	Gag (485‐497)	LTSLRSLF	1684	1.00	A*01:01			47	30	+	None
PQ16	PLTSLRSLFGNDPSSQ	C8166	Gag (485‐500)	LTSLRSLF	1684	1.00	A*01:01			68	37	+	60
SD9	SLRSLFGND	C8166	Gag (488‐496)	SLRSLFGN	17 464	32.00	B*08:01	KEMYPLASLRSLFGNDPSSQ	A1; Cw7	22		+	None
SQ13	SLRSLFGNDPSSQ	C8166	Gag (488‐500)	SLRSLFGNDPS	6984	10.00	B*08:01	KEMYPLASLRSLFGNDPSSQ	A1; Cw7	67	54	+	60
LQ12	LRSLFGNDPSSQ	C8166	Gag (489‐500)	LRSLFGNDPSS	26 385	32.00	C*07:01	KEMYPLASLRSLFGNDPSSQ	A1; Cw7	46	61	+	1470
RQ11	RSLFGNDPSSQ	C8166	Gag (490‐500)	RSLFGNDPS	23 586	32.00	C*05:01	KEMYPLASLRSLFGNDPSSQ	A1; Cw7	57	62	+	None
VY8	VLDVGDAY	C8166	Pol (263‐270)	VLDVGDAY	57	0.10	A*01:01	TVLDVGDAY	B*35;01	23		+	70
EW10	EELRQHLLRW	C8166	Pol (358‐367)	EELRQHLLRW	29	0.05	B*44:02	EELRQHLLRW	B44	48	37	+	None
DE11	DLVAEIQKQGE	C1866	Pol (479‐489)	AEIQKQGE	4606	3.00	B*44:02			24		‐	70
AW11	AEIQKQGQGQW	C8166	Pol (482‐492)	AEIQKQGQGQW	21	0.03	B*44:02			56	67	‐	940
AY13	AEIQKQGQGQWTY	C8166	Pol (482‐494)	AEIQKQGQGQW	21	0.03	B*44:02			68	78	+	620
YY17	YVDGAANRETKLGKAGY	C8166	Pol (596‐612)	RETKLGKAGY	216	0.40	B*44:02	RETKLGKAGY	A29	42	56	+	2140
SI9	SESELVNQI	C8166	Pol (668‐676)	SESELVNQI	67	0.12	B*44:02			35		+	None
LE8	LPPVVAKE	C8166	Pol (743‐750)	LPPVVAKE	45 152	50.00	A*01:01	LPPVVAKEI	B*51;01; B*07;02		32	na	None
QL10	QNVGKKLSKL	C1866	Pol (867‐876)	NVGKKLSKL	1452	3.00	B*08:01			28		+	70
SL9	SAEPVPLQL	C8166	Rev (67‐75)	SAEPVPLQL	469	0.80	C*05:01	SAEPVPLQL	B14, Cw8	39	57	+	None
GQ12	GTSGTQGVGSPQ	C8166	Rev (90‐101)	GTSGTQGV	14 242	7.00	A*01:01				21	na	None
SP9	SPQILVESP	C8166	Rev (99‐107)	SPQILVES	18 207	32.00	B*08:01				22	na	None
GW10	GVEMGHHAPW	C8166	Vpu (68‐77)	VEMGHHAPW	19	0.03	B*44:02			15		na	None
NY9	NFGPGGAIY	C1866	gp160 (310‐318)	NFGPGGAIY	12 517	6.00	A*01:01			29		+	None
YL8	YLKDQQLL	C8166	gp160 (586‐593)	YLKDQQLL	419	1.50	B*08:01	YLKDQQLL	A24; B8	23		na	260
NW11	NEQELLELDKW	C8166	gp160 (656‐666)	NEQELLELDKW	68	0.12	B*44:02			32		+	530
EL9	ELKNSAVSL	C8166	gp160 (806‐814)	ELKNSAVSL	392	1.00	B*08:01	QELKNSAVSL	B*40;01	16		na	5000
SR11	SYALASDAQNR	C8166	3′‐5′ frame 2 (1054‐1064)	SYALASDA	22 998	32.00	C*07:01			26		na	230

a HXB2: Position of the identified peptide sequence in the reference strain HXB2.

b Predicted binding segment: The segment of the identified sequence that has the highest probability to bind to either of the six alleles present in the regarding sample.

c Rank: Percentile rank, 2% defines the threshold for potential epitopes (90% sensitivity and 95% specificity) 20. Rank values above threshold are highlighted in green.

d Reported epitope: Reported epitope in LANL‐HIVDB.

e Reported HLA allele: HLA restriction previously reported in LANL‐HIVDB.

f PEAKS and MASCOT: Probability score: −10 × lg_10_(*p*) where *p* is the probability that the observed match is a true and not random event.

g SM: Comparison of synthetic peptide spectra and experimental spectra; “+” indicates a spectral match, “‐“indicates a mismatch.

h ELISPOT: Maximal response of 1/24 HIV‐1‐infected individuals screened for responses to the regarding peptide sequence in an ELISPOT assay (spot‐forming units/10^6^ PBMC). na: not analyzed.

For the primary infected cell line samples, sequence alignment of all identified 9‐mer peptides broadly confirmed an enrichment of the predicted amino acids in the anchor residue positions for the HLA allele genotype of the regarding sample (Fig. 1C) 17.

The eluted HIV‐1 peptides were of the following origins: 38 (51%) were from Gag; 9 (12%) were from Pol; 16 (21%) were from Env; 5 (7%) were from Nef; 3 (4%) were from Rev, and 3 (4%) were from Vpu (Fig. 1D and Table 1). Peptide SR11 (Table 1) originates from a protein translated from an alternative HIV‐1 reading frame 18. Overall, 21 (28%) peptides were derived from conserved regions of the HIV‐1 proteome (up to 6% amino acid variation), which are common to many HIV‐1 isolates and, therefore, attractive vaccine targets 19. Of the 75 identified HIV‐1 peptide sequences, only 13 (17%) matched previously identified optimal epitopes in the Los Alamos National Laboratory‐HIV Sequence Database (LANL‐HIVDB) and only 9/13 were previously reported for the HLA haplotypes of analyzed samples. For 27 (36%) peptides, either a longer sequence containing the identified peptide sequence or a fragment of the identified peptide was reported in LANL‐HIVDB, and for 18/27 sequences an HLA restriction matching one HLA allele of the haplotype analyzed was reported. A further 9 (12%) peptides were reported with one or two amino acid substitutions. The other 26 (35%) peptides were not previously mapped and were, therefore, considered novel. Notably, peptide FY9 was identified in all three primary infected T‐cell lines irrespective of the distinct HLA haplotypes and may be presented by a noncanonical HLA allele, i.e. HLA‐E.

**Figure 2:**
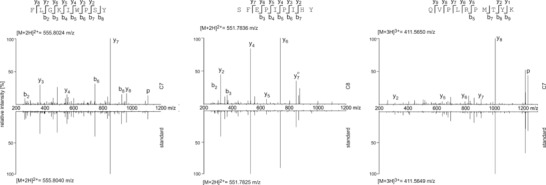
Spectral matches for HIV‐1‐derived peptides. HLA‐associated viral peptides sequences were synthesized and measured by LC‐MS/MS under identical conditions as the experimentally identified peptide sequences. Shown here are examples for one peptide sequence identified in each of the primary infected cell samples C6, C7, and C8. Both the experimental spectrum that was detected in the indicated sample and the spectrum acquired from the synthetic counterpart (standard) are plotted relative to each other to illustrate the spectral match. Fragment ions are labeled in the spectra and the regarding molecular fragment is indicated in the peptide sequence above each spectrum. Ions are labeled as follows: b: singly charged N‐terminal fragment ion; y: singly charged C‐terminal fragment ion; ^o^: loss of H_2_O; p: parent peptide ion. The detected mass to charge ratio [m/z] of the intact peptide parent ion is stated for each spectrum shown.

To experimentally affirm the correct assignment of the fragment spectra to precursor peptide sequences, identified HIV‐1‐derived peptides were synthesized and analyzed under identical conditions by LC‐MS/MS for spectrum matches. Of the 53 tested peptides, spectra of 48 (91%) could be confidently matched to those obtained from HIV‐1‐infected cells (Fig. 2, Table 1).

Using the NetMHCpan 2.8 MHC binding prediction algorithm and a percentile rank threshold of 2% 20, 21, 22, 23, 47 (62%) peptides were predicted to bind to one HLA allele expressed in the sample (Table 1). Generally, the prediction for binding of longer, nonstandard peptides is more challenging and limited to the identification of nested binding sequences. However, an extensive search of predicted nested binding sequences within the eluted peptide sequences increased the number of predicted binding sequences to 63 (84%).

### Peptide‐specific responses in HIV‐1‐infected individuals

**Figure 3:**
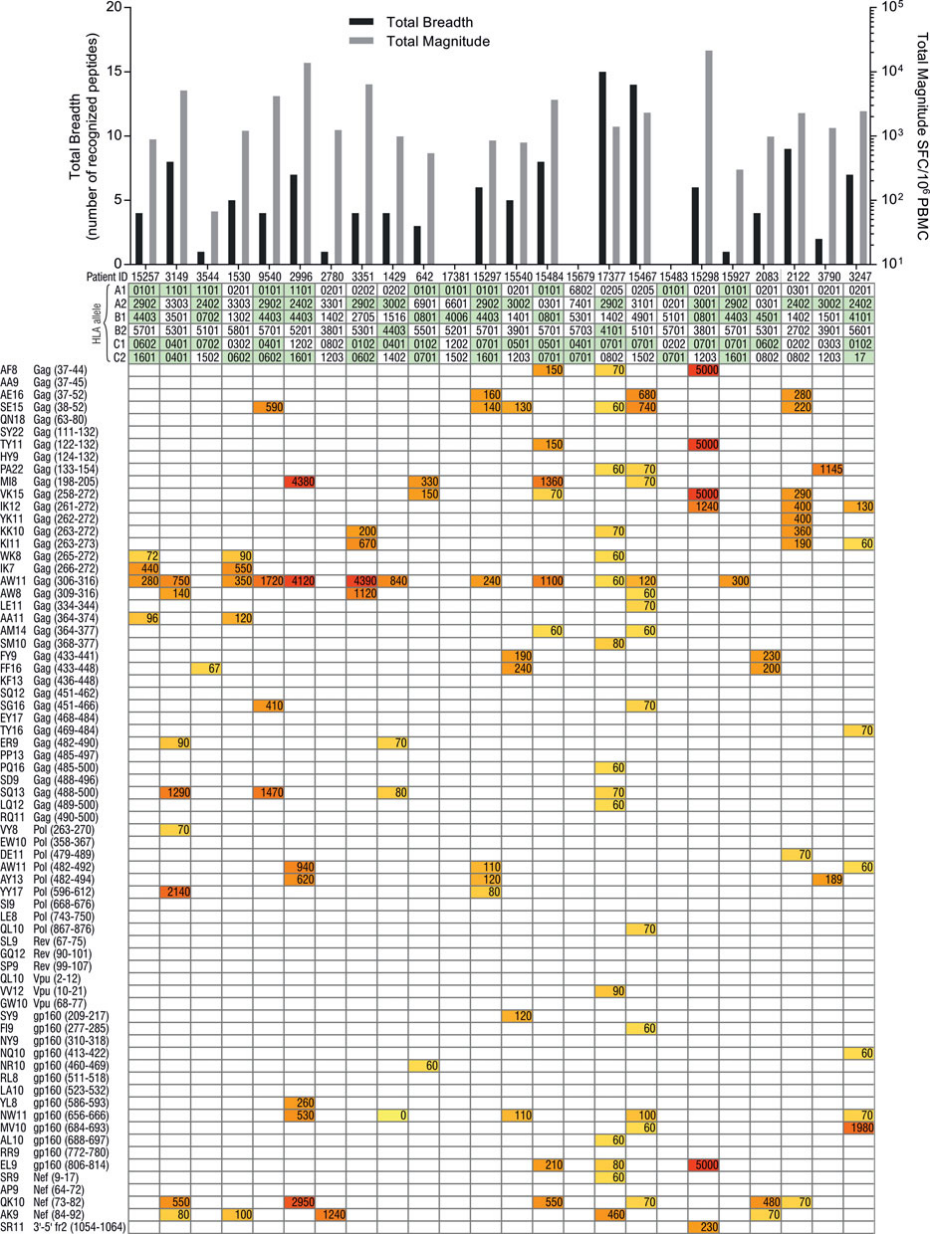
T‐cell responses to eluted HIV‐1 peptides identified in HIV‐1‐infected subjects. PBMCs from 24 HIV‐1 positive individuals were screened for T‐cell responses to the identified peptide sequences by determining IFN‐γ output in an ELISPOT assay. For each patient, the number of recognized peptides (breadth of the response) and the total magnitude of T‐cell responses are shown in the graph. The HLA genotype of all six class I alleles for HLA‐A, ‐B, and ‐C is given below each patient ID number. Alleles matching any of the alleles of the C8166 cell line or the three primary cell samples analyzed are highlighted in green. For reference, a heat map illustrating responses to each of the 70 peptide sequences tested is shown below for each patient.

A biological validation was performed by testing 70 peptides in interferon (IFN)‐γ ELISPOT assays for recognition by PBMCs from 24 HIV‐1‐infected subjects with variable HIV‐1 disease control. Careful selection of individuals ensured that all HLA alleles, from which HIV‐1 peptides were eluted, were covered. Overall, a median (range) of 4 (0–15) tested peptides were recognized per donor with a median (range) total magnitude of all added responses of 1225 (0–21 470) spot‐forming cells (SFC)/10^6^ PBMCs (Fig. 3). A total of 23/24 patients shared at least one HLA allele with the cells used for peptide elution and 21/24 individuals responded to at least one of the peptides, whereas only three subjects failed to respond to any peptide (Fig. 3). One individual without any HLA match still showed recognition of one stimulatory peptide, likely responding through alleles belonging to different HLA supertype as described previously 24, 25 or through CD4^+^ T‐cell recognition. The median (range) number of peptides recognized per individual was 4.9 (0–15) and 23/70 (32%) tested peptides were not recognized by any individual (with or without matching HLA allele). Peptide AW11 (Gag) was recognized by 12/24 subjects; this is an optimal epitope described in the LANL‐HIVDB restricted by HLA‐C*05:01.

The rest of the eluted peptides were recognized between one and six times. Importantly, most of the observed responses targeted peptides that had not previously been defined as epitopes, and only a small proportion of responses were specific for known optimal epitopes. Of the peptides that did not match any HLA‐binding motif of the corresponding cell line, 50% (6/12) gave T‐cell responses, in contrast to a 71% (41/58) response rate for peptides that did match at least one HLA‐binding motif. For peptides with predicted binding stronger than or equal to 0.1%, 83% (10/12) gave a T‐cell response.

## Discussion

Changes in HLA‐associated peptide presentation have been analyzed in the context of HIV‐1 infection 26 and more recently, HIV‐1‐specific, HLA‐associated peptides have been identified in a model cell line secreting HLA‐A*11:01 molecules 11, 26, providing critical novel information for the definition of T‐cell targets in HIV‐1 infection. However, the ability to define HLA‐class I‐associated, HIV‐1‐derived peptidome directly from HIV‐1‐infected primary cells allows a more precise view of the peptides presented on HIV‐1‐infected cells and facilitates detection of natural T‐cell targets.

In our analyses, 35% of eluted HIV‐1‐derived peptides had not to date been reported to be presented by HLA class I and 83% of the peptides had not been previously mapped to a precise HLA‐binding sequence. Underrepresentation of Pol‐derived peptides from primary infected CD4^+^ cells was notable and concurred with some previous reports 27, 28, 29, these peptides may be less abundantly presented on HLA‐I molecules in infected cells compared to Gag‐derived peptides. However, the number of vaccine‐elicited Pol‐specific CD8^+^ effectors has been shown to correlate with inhibition of HIV‐1 replication in autologous cells at least equally to Gag‐specific responses 30, 31. Thirty‐two percent of the eluted peptides were not recognized in subjects used in this study that were naturally infected with HIV‐1. This could be because the viruses with which these individuals were infected deviated from the relevant sequences, or because responses mounted to these peptides had declined to undetectable frequencies at the time of sampling due to viral escape. Alternatively, responses to these peptides may be subdominant to undetectable levels. On the other hand, responses to some peptides may also not have been induced due to a gap in T‐cell repertoire or due to HLA restriction. Nevertheless, vaccination may induce T‐cell responses against these peptides and therefore these sequences remain to be useful targets for T‐cell vaccination.

In conclusion, we demonstrate here that we now have the capacity to delve more deeply into the HLA class I‐associated immunopeptidome of primary infected cells to identify less‐abundant pathogen‐derived peptides. These advances bring us one step further toward identification of T‐cell targets on primary cells isolated from infected individuals in a clinical setting which will be of exceptional importance for the development of personalized immune treatments.

## Materials and methods

### HIV‐1 IIIB virus stock preparation

HIV‐1 IIIB (clade B, CXCR4‐tropic) isolate was obtained from the Program EVA Centre for AIDS Reagents, National Institute for Biological Standards and Control (NIBSC) and expanded as described previously 12. HIV‐1 IIIB viral stocks were prepared by propagation in primary CD4^+^ cells and virus‐containing supernatant was harvested at day 6 postinfection, aliquoted, and frozen at −80°C. Fifty percent tissue culture infectious dose (TCID_50_) was calculated as described previously 12.

### Cell culture

C8166 cells or CD4^+^ T cells purified from PBMC by magnetic bead selection were stimulated with phytohemagglutinin (5 μg/mL) in RPMI‐1640 medium supplemented with 10% FCS (R10) for 3 days, washed, and infected with HIV‐1 IIIB at a MOI of 0.01. This preselected MOI yielded detectable infection in all wells when tested in the TCID_50_ assay without causing significant cell death, i.e. less 20% lymphocytes stained with Aqua Live/Dead (Invitrogen, data not shown). Infection was achieved by spinoculation for 2 h at 25°C, after which cells were washed twice and cultured at 1.5 × 10^6^ cells/mL in R10 supplemented with IL‐2 (20 IU/mL) for 5–7 days before harvesting the cells. To estimate the percent infection, 0.5 × 10^6^ cells were harvested and stained first with Aqua Live/Dead Fixable stain (Invitrogen), fixed with 4% paraformaldehyde solution/lysolecithin (20 μg/mL) at room temperature and resuspended in cold 50% methanol for 15 min. Further permeabilization was achieved with 0.1% Nonidet P‐40 and cells were then stained with antibodies to HIV‐1‐ Gag p24 (KC‐57‐FITC, Beckman Coulter) followed by antibodies to CD3, CD4, and CD8 conjugated to APC‐Cy7, PerCP, and APC, respectively (BD Biosciences). Samples were acquired on a CyAn flow cytometer and analyzed using FlowJo (version 9.2). If the infection rates were equal or above 20% of CD4^+^CD3^+^ live cells, cells were harvested and lysed using cell lysis buffer (1% Igepal 630, 300 mM NaCl, 100 mM Tris pH 8.0). Intracellular p24 was detected after NP‐40 permeabilization and staining with a HIV‐1 Gag p24‐specific antibody (KC‐57).

### HLA class I immunoprecipitation and HPLC fractionation

Purification of HLA class I‐bound peptides was carried out as previously described 32. Briefly, lysates of infected cells were cleared by two subsequent centrifugation steps at 500 × *g* for 10 min and 20.000 × *g* for 30 min. HLA complexes were captured on Protein A‐sepharose beads (Expedeon) cross‐linked to W6/32 antibody (5 mg/mL) 32 at gravity flow and washed using subsequent runs of 50 mM Tris buffer, pH 8.0 containing first 150 mM NaCl, then 400 mM NaCl, and then, no salt. HLA‐peptide complexes were eluted with 5 mL 10% acetic acid. Affinity column‐eluted material was loaded onto on a 4.6 × 50 mm ProSwift RP‐1S column (Thermo Fisher Scientific) and eluted using a 500 μL/min flow rate over 10 min from 2 to 35% buffer B (0.1% formic acid in acetonitrile) in buffer A (0.1% formic acid in water) using an Ultimate 3000 HPLC system (Thermo Scientific). Detection was performed using a variable wavelength detector at 280 nm. Fractions up to 12 min that did not contain ß_2_‐microglobulin were combined and dried.

### LC‐MS/MS analysis

Each sample was resuspended in 20 μL buffer A and analyzed both on an Orbitrap Elite (Thermo Scientific) online coupled to an Acquity nano UPLC (Waters) and a TripleTOF 5600 (AB SCIEX) coupled to an Eksigent ekspert nanoLC 400 cHiPLC system. *Orbitrap Elite*: Peptides were separated on a nano Acquity UPLC system (Waters) supplemented with a 25 cm BEH130 C18 column, 1.7‐mm particle size using a linear gradient from 8 to 35% buffer B in buffer A at a flow rate of 250 nL/min for 60 min. Peptides were introduced to an Orbitrap Elite mass spectrometer using a nanoESI source. Subsequent isolation and collision‐induced dissociation was induced on the 20 most abundant ions per full MS scan using an isolation width of 1.5 amu. All fragmented precursor ions were actively excluded from repeated selection for 15 s. *TripleTOF 5600*: Peptides were separated on an ekspert nanoLC 400 cHiPLC system (Eksigent) supplemented with a 15 cm x 75 μm ChromXP C18‐CL, 3 μm particle size using a linear gradient from 8% buffer A to 35% buffer B at a flow rate of 300 nL/min for 60 min. Peptides were introduced to TripleTOF 5600 mass spectrometer and collision‐induced dissociation fragmentation using ramped collision energy was induced on the 30 most abundant ions per full MS scan using unit isolation width 0.7 amu. All fragmented precursor ions were actively excluded from repeated selection for 15 s.

### MS data analysis interpretation

Raw data were converted to MASCOT generic files using msconvert 33 or ProteinPilot 4.5 34. Sequence interpretation of MS/MS spectra were performed using a database containing all annotated human SwissProt entries including translations of all six reading frames of the sequenced HIV‐1 IIIB genome in addition to translations of all known assigned HIV‐1 protein coding regions (GenBank KJ925006) or a database containing all annotated human SwissProt entries (02/2013, 20 253 entries) and all HIV‐1 entries in NCBI (02/2013, 446 954 entries) with PEAKS 7 33 and MASCOT 2.4 34, 35. The probability score threshold was defined by decoy database searches implemented in the regarding search engines at a general false discovery rate of 5%.

### Ethics statement

Chronically HIV‐1‐infected individuals were recruited from the HIV Unit in Hospital Germans Trias i Pujol, Badalona (*n* = 16) and Hospital de la Vall d'Hebron, Barcelona, Spain (*n* = 8). The study was approved by the Institutional Review Board of both participating hospitals and all individuals provided written informed consent before entering the study. PBMC samples were drawn and processed within 4 h after venipuncture and the cells were stored in liquid nitrogen until use.

### IFN‐γ ELISPOT assay

IFN‐γ ELISPOT assay was performed as previously described 24, 36. A screening for CTL responses was developed using a matrix of 70 eluted peptides from immunoprecipitated HLA class I complexes. Cryopreserved PBMCs from 24 subjects were incubated with the matrix peptide pools in a precoated plate (Millipore, Barcelona, Spain) with anti‐human IFN‐γ monoclonal antibody (Mabtech, Sweden). Cells with R10 medium only were used as negative controls and cells with phytohemagglutinin were used as positive controls. PBMCs were cultured overnight at 37°C, 5% CO_2_ atmosphere, and then washed six times with PBS. The plates were then incubated for 1 h at room temperature with the biotinylated anti‐I IFN‐γ monoclonal antibody (Mabtech) followed by six washes and 1 h incubation with the streptavidin‐coupled alkaline phosphatase (Mabtech). After washing the plate, nitro blue tetrazolium and 5‐bromo‐4‐chloro‐3‐indolul phosphate (Bio‐Rad, Barcelona, Spain) were added for color development. After a short incubation, the reaction was stopped by washing the plate with tap water. The IFN‐γ production was detected as blue spots on the membrane, the spot‐forming units were counted with an automated ELISPOT reader system (CTL, Germany) using ImmunoSpot software package. Responses were defined as positive if they exceeded (i) 50 spot‐forming units/10^6^ PBMC per well, (ii) the mean of negative wells plus three standard deviations, and (iii) three times the mean of the negative well, whichever was higher.

## Conflict of interest

The authors declare no financial or commercial conflict of interest.

**Abbreviations**
MS: mass spectrometry
LC‐MS/MS: liquid chromatography tandem mass spectrometry
AIDS: acquired immunodeficiency syndrome
LANL‐HIVDB: Los Alamos National Laboratory‐HIV Sequence Database

## Supporting information

As a service to our authors and readers, this journal provides supporting information supplied by the authors. Such materials are peer reviewed and may be re‐organized for online delivery, but are not copy‐edited or typeset. Technical support issues arising from supporting information (other than missing files) should be addressed to the authors.

Peer review correspondence
