# Supplementary material for: Defining the HLA class I‐associated viral antigen repertoire from HIV‐1‐infected human cells
Source: Eur J Immunol. 2015 Nov 4;46(1):60–9. doi: 10.1002/eji.201545890 (PMC4737398; doi:10.1002/eji.201545890)
Supplement: Supplementary file 1 — Peer review correspondence [file EJI-46-60-s001.pdf]

Peer review correspondence

European Journal of Immunology

Defining the HLA class I-associated viral antigen repertoire from HIV-1-infected human cells

Nicola Ternette, Hongbing Yang, Thomas Partridge, Anuska Llano, Samandhy Cedeño, Nadine L. Dudek, Beatriz Mothe, Manuel Crespo, William M. Fischer, Bette T.M. Korber, Morten Nielsen, Persephone Borrow, Anthony W. Purcell, Christian Brander, Lucy Dorrell, Benedikt M. Kessler and Tomáš Hanke

Corresponding author: Nicola Ternette, TDI Mass Spectrometry Laboratory, Nuffield Department of Medicine, University of Oxford, Oxford, United Kingdom

---

Review Timeline:	Submission date:	2 July 2015
	Editorial decision:	28 July 2015
	Revision received:	27 August 2015
	Accepted:	09-October 2015

Handling Executive Committee member: Prof. Annette Oxenius

Please note that the correspondence below does not include the standard editorial instructions regarding preparation and submission of revised manuscripts, only the scientific revisions requested and addressed.

First Editorial Decision – 28 July 2015

Dear Dr. Ternette,

Manuscript ID eji.201545890 entitled "Definition of the HLA class I-associated viral antigen repertoire from HIV-1-infected cells" which you submitted to the European Journal of Immunology has been reviewed. The comments of the referee are included at the bottom of this letter.

A revised version of your manuscript that takes into account the comments of the referee will be reconsidered for publication.

You should also pay close attention to the editorial comments included below. **In particular, please edit your figure legends to follow Journal standards as outlined in the editorial comments. Failure to do this will result in delays in the re-review process.**

Please note that submitting a revision of your manuscript does not guarantee eventual acceptance, and that your revision will be re-reviewed by the referee before a decision is rendered.

If the revision of the paper is expected to take more than three months, please inform the editorial office. Revisions taking longer than six months may be assessed by new referee to ensure the relevance and timeliness of the data.

Once again, thank you for submitting your manuscript to European Journal of Immunology and we look forward to receiving your revision.

Yours sincerely,
Katharina Schmidt

On behalf of Prof. Annette Oxenius

Dr. Katharina Schmidt
European Journal of Immunology
e-mail: ejied@wiley.com
www.eji-journal.eu

****

Reviewer: 1

Comments to the Author
Definition of the HLA class I-associated viral antigen repertoire from HIV-1-infected cells

The authors characterised HLA class I ligands extracted from HIV-infected cells and analysed the immune response of infected donors to these peptides by IFNg ELISPOT.

This is an elaborate study giving for the first time insight into the immunopeptidome of HIV. It is acknowledged that primary cells have been included. Results are presented in a comprehensive manner, so the paper should benefit the society in this field. There are, however, some conclusions that are not fully justified by the experimental data. The followings are some comments for potential improvement of the manuscript.

1. Of the 75 sequences shown in Table 1, a significant proportion does not fulfil the quality criteria for classification as HLA class I ligands. 26 sequences show unusual length (< 8 aa or > 12 aa) and/or unusual C-terminal aa (polar residues such as E, S, or Q are never found in HLA class I motifs). Therefore, these peptides most probably do not “originate from HLA complexes in the ER”, but merely are

contaminations carried through the one-step purification procedure. The authors claim that 80-90% of the peptides were 8-12 amino acids long – the percentage of peptides of correct length in Table I is much lower. Obviously, among the human self peptides identified, similar artefacts have not been identified to such an extent. There is no use to assign “predicted binding segments” within these sequences. In order to improve this report, the authors should remove all conspicuous artefacts which do not clearly show features of HLA class I ligands.

2. Immune responses were tested by ELISPOT assays using PBMCs. Therefore, neither HLA association nor contribution of CD8+ cells are proven. Responses to long peptides may well be caused by CD4+ T cells. So it might be more appropriate to elute HLA class II ligands and/or to test responses by more specific FACS assays or by using purified T-cell populations. Such experiments are not required for this paper but the authors should more thoroughly discuss about the pitfalls of their approach.

3. It should be mentioned how many replicates of extractions and LC/MSMS analyses (all with 10e8 cell equivalents?) have been carried out. It should also be pointed out how human sequences were discriminated from HIV sequences by the software and data interpretation.

4. I would be interesting to know the HIV strains (including sequence variants of respective T-cell epitopes) the donors are infected with. This might facilitate the discussion on recognition/non-recognition of peptides. On the other hand, do all peptides presented by class I molecules have to be recognised by T cells? The authors should consider the concept of immunodominance.

5. There are some errors in Fig. 1 C with respect to the peptide motifs. B*08:01 does not require K in position 2, the E in P2 of A*29:02 has long been falsified (see also FY9), and B*27:05 accepts basic residues in P9 in addition to hydrophobic aa.

<u>First revision – authors’ response – 27 August 2015</u>

Comments to Reviewer #1

"The authors characterised HLA class I ligands extracted from HIV-infected cells and analysed the immune response of infected donors to these peptides by IFNg ELISPOT."

"This is an elaborate study giving for the first time insight into the immunopeptidome of HIV. It is acknowledged that primary cells have been included. Results are presented in a comprehensive manner, so the paper should benefit the society in this field. There are, however, some conclusions that are not

fully justified by the experimental data. The followings are some comments for potential improvement of the manuscript."

"1. Of the 75 sequences shown in Table 1, a significant proportion does not fulfil the quality criteria for classification as HLA class I ligands. 26 sequences show unusual length (< 8 aa or > 12 aa) and/or unusual C-terminal aa (polar residues such as E, S, or Q are never found in HLA class I motifs). Therefore, these peptides most probably do not “originate from HLA complexes in the ER”, but merely are contaminations carried through the one-step purification procedure."

It is correct that the majority of peptides bound to HLA class I has been shown to be in the range of 8-12 amino acids. However, the observation of longer peptides (up to 25 amino acids) bound to MHC class I has been reported before in the context of some HLA alleles (described in references 14-16). The characterisation of peptides bound to secreted HLA A*11:01 from HIV-1 infected cells also showed occurrence of longer ligands (reference 11).

"The authors claim that 80-90% of the peptides were 8-12 amino acids long – the percentage of peptides of correct length in Table I is much lower."

These percentages results from analysis of the complete dataset including all human sequences (self-peptides) that were identified in this sample set. We corrected the numbers stated in the manuscript to the more accurate values 78-92 percent, please see page 6.

"Obviously, among the human self peptides identified, similar artefacts have not been identified to such an extent. There is no use to assign “predicted binding segments” within these sequences. In order to improve this report, the authors should remove all conspicuous artefacts which do not clearly show features of HLA class I ligands."

We agree that there is no evidence that these peptides bind through an internal binding segment or whether the binding occurs via a bulged central part of the peptide, or not at all. Similar experiments with cells that do not express MHC class I (.221 cells) in our hands do not give rise to any significant background peptides.
Therefore, we rather prefer an unbiased presentation of the data without filtering for an asumed outcome and refer to our comment above that longer peptides have previously been characterised as bound to MHC class I molecules.
Also, since our biochemical purification procedure may also retrieve not yet fully processed MHC class I bound ligands from the endoplasmatic reticulum, we feel that an attempt to assign a possible internal binding segment is interesting as it results in the explanation of binding in 12 out of 17 longer peptide sequences.

European Journal of Immunology

"2. Immune responses were tested by ELISPOT assays using PBMCs. Therefore, neither HLA association nor contribution of CD8+ cells are proven. Responses to long peptides may well be caused by CD4+ T cells. So it might be more appropriate to elute HLA class II ligands and/or to test responses by more specific FACS assays or by using purified T-cell populations. Such experiments are not required for this paper but the authors should more thoroughly discuss about the pitfalls of their approach."

We thank the reviewer for this comment. The majority of responses measured in our ELISPOT assays were previously shown to be mediated by CD8+ T cells (Frahm et al. 2004), now referenced in the methods section [36]. In order to improve clarity on the presented data, we have extended figure 3. The figure now shows the responses of each patient to each of the identified peptide sequences.

"3. It should be mentioned how many replicates of extractions and LC/MSMS analyses (all with 10e8 cell equivalents?) have been carried out."

We thank the reviewer for pointing out that this information was not stated clearly in the manuscript. Each sample (C6,C8,C7, C8166) was analysed by both an Orbitrap Elite and Triple TOF 5600 mass spectrometry instrument as now clearly stated in the revised methods section (page 11). Also, the exact percentage of infection of the approximately 10e8 cells infected for each sample is now given in the results section (page 5).

"It should also be pointed out how human sequences were discriminated from HIV sequences by the software and data interpretation."

The analysis of peptide sequences using LC-MS/MS is based on intact peptide mass measurement (MS) followed by fragment mass measurement (MS/MS) that allows the determination of the complete or partial amino acid sequence of the peptide. Comparison with the described database of combined human and viral sequences can then identify the exact originating peptide sequence for each measured MS/MS spectrum, which can be of human or viral origin. The data interpretation is validated by random database searches and a threshold of 5% false discovery rate was chosen in this study.

"4. I would be interesting to know the HIV strains (including sequence variants of respective T-cell epitopes) the donors are infected with. This might facilitate the discussion on recognition/non-recognition of peptides. On the other hand, do all peptides presented by class I molecules have to be recognised by T cells? The authors should consider the concept of immunodominance."

We agree with the reviewer that it would be very interesting to know the sequences of the donors included in this study. Sequencing of all HIV-1 strains of analysed infected individuals was unfortunately beyond the scope of this study that aimed to validate the sequences by recognition from HIV-1-infected donors.

Truly, the concept of immunodominance and T cell repertoire should be mentioned as suggested by the reviewer. We have now expanded the discussion: 'This could be because the viruses with which these individuals were infected deviated from the relevant sequences, or because responses mounted to these peptides had declined to undetectable frequencies at the time of sampling due to viral escape. Alternatively, responses to these peptides may be subdominant to undetectable levels. On the other hand, responses to some peptides may also not have been induced due to a gap in T-cell repertoire or due to HLA restriction.' (page 9)

"5. There are some errors in Fig. 1 C with respect to the peptide motifs. B*08:01 does not require K in position 2, the E in P2 of A*29:02 has long been falsified (see also FY9), and B*27:05 accepts basic residues in P9 in addition to hydrophobic aa."

We apologize for the errors in the known motifs for human HLA alleles. The database used (SYFPEITHY) was not updated in a long time. We have corrected all motifs in figure 1C using sequence motifs from the MHC Motif Viewer (www.cbs.dtu.dk/biotools/MHCMotifViewer). For a clearer synopsis, C alleles that are expressed at low levels and do contribute to the motif to a very minor extend, were removed from the overview (see figure 1 and legend).

<u>Second Editorial Decision – 5 October 2015</u>

Dear Dr. Ternette,

It is a pleasure to provisionally accept your manuscript entitled "Definition of the HLA class I-associated viral antigen repertoire from HIV-1-infected cells" for publication in the European Journal of Immunology. For final acceptance, please follow the instructions below and return the requested items as soon as possible as we cannot process your manuscript further until all items listed below are dealt with.

Please note that EJI articles are now published online a few days after final acceptance (see Accepted Articles: http://onlinelibrary.wiley.com/journal/10.1002/(ISSN)1521-4141/accepted). The files used for the Accepted Articles are the final files and information supplied by you in Manuscript Central. You should therefore check that all the information (including author names) is correct as changes will NOT be permitted until the proofs stage.

Peer review correspondence

We look forward to hearing from you and thank you for submitting your manuscript to the European Journal of Immunology.

Yours sincerely,
Karen Chu

on behalf of Prof. Annette Oxenius

Dr. Karen Chu
Editorial Office
European Journal of Immunology
e-mail: ejied@wiley.com
www.eji-journal.eu
